# General rules for clinical and pathological studies on oral cancer (2nd edition): a synopsis

**DOI:** 10.1007/s10147-020-01812-9

**Published:** 2021-03-15

**Authors:** Yoshihide Ota, Tadahide Noguchi, Eiichiro Ariji, Chihiro Fushimi, Nobukazu Fuwa, Hiroyuki Harada, Takafumi Hayashi, Ryuichi Hayashi, Yoshitaka Honma, Masahiko Miura, Taisuke Mori, Hitoshi Nagatsuka, Masaya Okura, Michihiro Ueda, Narikazu Uzawa, Kazuhiro Yagihara, Hisao Yagishita, Masashi Yamashiro, Souichi Yanamoto, Tadaaki Kirita, Scientific Committee on General Rules for Clinical and Pathological Studies of Oral Cancer, Japanese Society of Oral Oncology

**Affiliations:** 1 Division of Surgery, Department of Oral and Maxillofacial Surgery, Tokai University School of Medicine, 143 Shimokasuya, Isehara, Kanagawa 259-1193 Japan; 2 Department of Dentistry, Oral and Maxillofacial Surgery, Jichi Medical University, 3311-1 Yakushiji, Shimotsuke, Tochigi 329-0498 Japan; 3 Department of Oral and Maxillofacial Radiology, Aichi-Gakuin University School of Dentistry, 2-11 Suemori-dori, Chikusa-ku, Nagoya, 464-8651 Japan; 4 Department of Head and Neck Oncology and Surgery, International University of Health and Welfare Mita Hospital, 1-4-3 Mita, Minato-ku, Tokyo, 108-8329 Japan; 5 Department of Radiation Oncology, Ise Red Cross Hospital, 1-471-2 Funae, Ise, Mie 516-8512 Japan; 6 Department of Oral and Maxillofacial Surgery, Graduate School of Medical and Dental Sciences, Tokyo Medical and Dental University, 1-5-45 Yushima, Bunkyo-ku, Tokyo, 113-8549 Japan; 7 Division of Oral and Maxillofacial Radiology, Niigata University Graduate School of Medical and Dental Sciences, 2-5274 Gakkocho-dori, Chuo-ku, Niigata, 951-8514 Japan; 8 Department of Head and Neck Surgery, National Cancer Center Hospital East, 6-5-1 Kashiwanoha, Kashiwa, Chiba 277-8577 Japan; 9 Head and Neck Medical Oncology Division, National Cancer Center Hospital, 5-1-1 Tsukiji, Chuo-ku, Tokyo, 104-0045 Japan; 10 Department of Oral Radiation Oncology, Graduate School of Medical and Dental Sciences, Tokyo Medical and Dental University, 1-5-45 Yushima, Bunkyo-ku, Tokyo, 113-8549 Japan; 11 Diagnostic Pathology Division, National Cancer Center Hospital, Tsukiji 5-5-1, Chuo-ku, Tokyo, 104-0045 Japan; 12 Department of Oral Pathology and Medicine, Graduate School of Medicine Dentistry and Pharmaceutical Sciences, Okayama University, 2-5-1 Shikata-cho, Kita-ku, Okayama, 700-8525 Japan; 13 Department of Dentistry and Oral Surgery, Saiseikai Matsuzaka General Hospital, 1-15-6 Asahi-cho, Matsuzaka, Mie 515-8557 Japan; 14 Department of Clinical Oral Oncology, Hokkaido Cancer Center, 2-3-54 Kikusui 4, Shiroishi-ku, Sapporo, Hokkaido 003-0804 Japan; 15 Department of Oral and Maxillofacial Surgery II, Graduate School of Dentistry, Osaka University, 1-8 Yamadaoka, Suita, Osaka 565-0871 Japan; 16 Department of Oral Surgery, Saitama Cancer Center, 780 Komuro, Ina-machi, Kitaadachi-gun, Saitama, 362-0806 Japan; 17 Division of Oral Diagnosis, Dental and Maxillofacial Radiology and Oral Pathology Diagnostic Services, The Nippon Dental University Hospital, 1-9-20 Fujimi, Chiyoda-ku, Tokyo, 102-8159 Japan; 18 Department of Dentistry and Oral Surgery, NTT Medical Center Tokyo, 5-9-22 Higash-Gotanda, Shinagawa-ku, Tokyo, 141-8625 Japan; 19 Department of Clinical Oral Oncology, Graduate School of Biomedical Sciences, Nagasaki University, 1-7-1 Sakamoto, Nagasaki, 852-8588 Japan; 20 Department of Oral and Maxillofacial Surgery, School of Medicine, Nara Medical University, 840 Shijo-cho, Kashihara, Nara 634-8521 Japan

**Keywords:** Oral cancer, General rules, Clinical and pathological studies

## Abstract

For doctors and other medical staff treating oral cancer, it is necessary to standardize the basic concepts and rules for oral cancer to achieve progress in its treatment, research, and diagnosis. Oral cancer is an integral part of head and neck cancer and is treated in accordance with the general rules for head and neck cancer. However, detailed rules based on the specific characteristics of oral cancer are essential. The objective of this article was to contribute to the development of the diagnosis, treatment, and research of oral cancer, based on the correct and useful medical information of clinical, surgical, pathological, and imaging findings accumulated from individual patients at various institutions. Our general rules were revised as the UICC was revised for the 8th edition and were published as the Japanese second edition in 2019. In this paper, the English edition of the “Rules” section is primarily presented.

## Introduction

Oral cancer is an integral part of head and neck cancer and is treated in accordance with the general rules for head and neck cancer. A part of the rules for managing oral cancer overlaps with the “General Rules for Clinical and Pathological Studies on head and neck cancer” published in 2018 (6th edition, Tokyo, Kanehara & Co. Ltd.), which can be said to differ from the general rules for cancers of other organs. However, the oral cavity is unique, since it contains the tongue, mandible, and teeth, with corresponding multiple and diverse functions such as swallowing, articulation, occlusion, and mastication. Moreover, the placement of various importance structures, such as nerves, blood vessels, and major salivary gland ducts is responsible for the anatomical complexity of the oral cavity. Therefore, we authored the “General Rules for Clinical and Pathological Studies on Oral Cancer” [1]. These rules were formulated, in principle, on the basis of the UICC and WHO classifications, but were partially revised when revision was considered necessary, and were proposed as a draft by the Japanese Society of Oral Oncology. We have devised detailed and highly accurate rules for the management of oral cancer, in order to overcome these problems. We referred to the contents of the general rules for head and neck cancer to ensure consistency for both rules.

Our general rules were revised as the UICC was revised for the 8th edition and were published as the second edition in Japanese in 2019 [2]. The revision consisted of 3 parts, i.e., the rules, explanations, and references, totaling 169 pages. In this paper, the English edition of the “Rules” section is primarily presented because of a limitation of space.

## Objectives

In order to enable people handling oral cancer in various positions to be able to conduct diagnosis, treatment, and research uniformly, the standardization of basic concepts and specific methods for their handling is necessary. The objectives of the rules listed in this article were to collect clinical, surgical, and pathological findings, including image information, by employing common standards; to clarify detailed pathological features; to accumulate useful medical information from individual patients at various facilities; and to contribute to the development of diagnosis, treatment, and research of oral cancer.

## Target diseases

In the present rules, oral cancer means carcinoma originating in the covering mucosa of the lip and six sites in the oral cavity, according to the UICC classification. The sites include cancer of the lip, buccal mucosa, upper gingiva, lower gingiva, hard palate, tongue, and floor of the mouth, in order of incidence. Secondary cancer is excluded. Cancer of the minor salivary gland and oral mucosal melanoma are included, while cancer of the major salivary gland is excluded.

## Rules

### I. Clinical findings

#### (1) Primary lesion

##### Anatomical sites and subsites

**Fig. 1:**
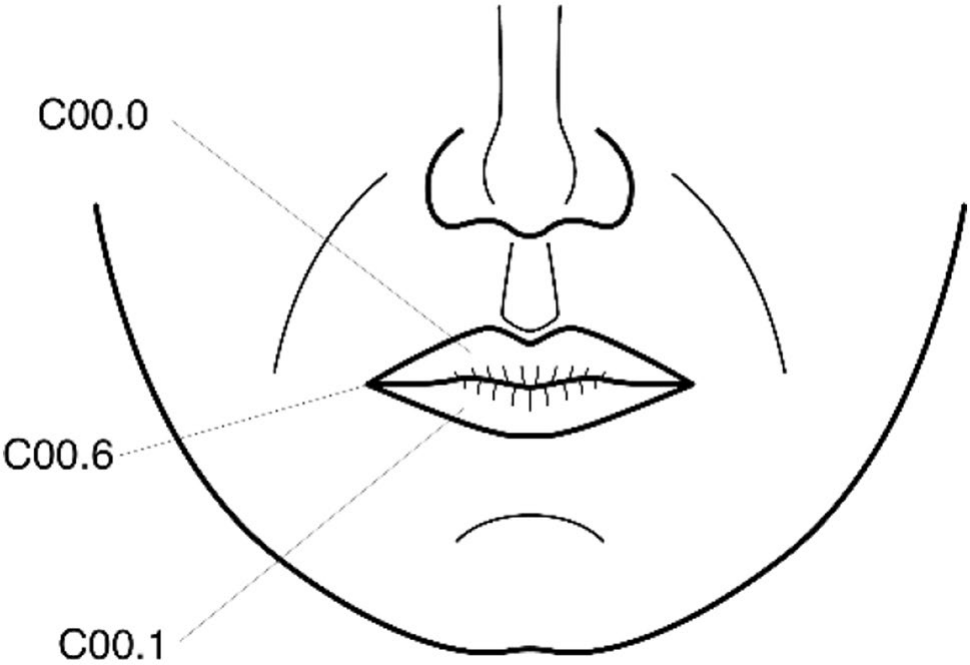
Anatomical sites and subsites of the lip

**Fig. 2:**
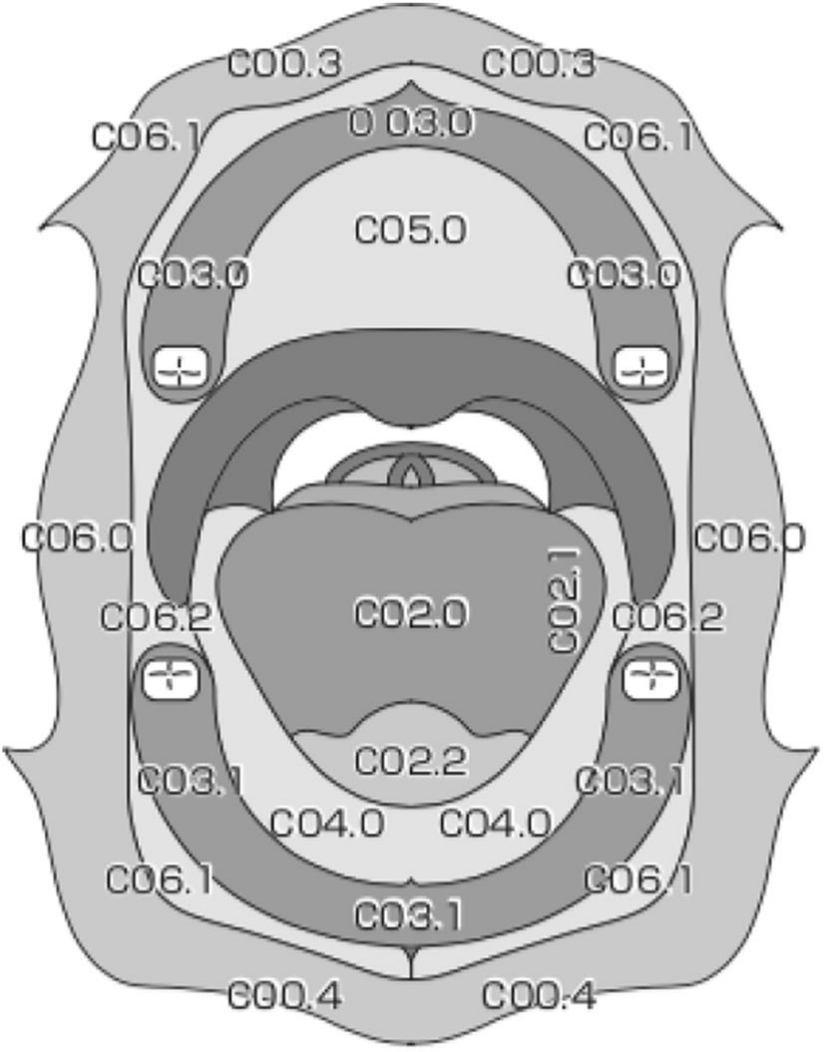
Anatomical sites and subsites of the oral cavity

- Lip (C00) (Fig. 1).

  1. External upper lip (vermilion border) (C00.0).
  2. External lower lip (vermilion border) (C00.1).
  3. Commissures (C00.6).
- Oral Cavity (CO2-006) (Fig. 2).

  1. Buccal mucosa:
    - Mucosa of upper and lower lips (C00.3, 4);
    - Cheek mucosa (C06.0);
    - Retromolar areas (C06.2);
    - Buccoalveolar sulci, upper and lower (vestibule of mouth) (C06.1).
  2. Upper alveolus and gingiva (upper gum) (C03.0).
  3. Lower alveolus and gingiva (lower gum) (C03.1).
  4. Hard palate (C05.0)
  5. Tongue
    - Dorsal surface and lateral borders anterior to vallate papillae (anterior two-thirds) (C02.0, 1);
    - Inferior (ventral) surface (C02.2).
  6. Floor of mouth (C04.0)

##### Size

(Long diameter) × (Short diameter) × (Thickness) mm.

Number of lesions (single/multiple).

- Measure the size of the part diagnosed to be cancer by inspection and palpation. The premalignant lesions, such as leukoplakia, that continue to the lesion are described separately. Submucosal induration is measured, including the area diagnosed with cancer.

##### Depth of invasion (DOI)

The depth of invasion (DOI) has been added as a modification to T factors. The DOI concept applies only to carcinomas arising in the lip and oral cavity. DOI is measured from the level of the basement membrane of the closest adjacent normal mucosa. A “plumb line” is dropped from this place to the deepest portion of cancer invasion. Thus, it is important to recognize the distinction between tumor thickness and true DOI. Clinically, DOI is categorized as being less than or equal to 5 mm, greater than 5 mm but not greater than 10 mm, and greater than 10 mm.

- Different from the DOI used in the digestive tract.

##### Macroscopic types

- Superficial type: Those primary showing superficial growth;
- Exophytic type: Those primarily showing exophytic growth;
- Endophytic type: Those primarily showing endophytic growth.

##### T factor

TX: Primary tumor cannot be assessed.
T0: No evidence of primary tumor.
Tis: Carcinoma in situ.
T1: Tumor 2 cm or less in greatest dimension and 5 mm or less depth of invasion.
T2: Tumor 2 cm or less in greatest dimension and more than 5 mm depth of invasion or tumor more than 2 cm but not more than 4 cm in greatest dimension and depth of invasion no more than 10 mm.
T3: Tumor more than 2 cm but not more than 4 cm in greatest dimension and depth of invasion more than 10 mm or tumor more than 4 cm in greatest dimension and not more than 10 mm depth of invasion.
T4a (Lip): Tumor invades through cortical bone, inferior alveolar nerve, floor of mouth, skin (of the chin or the nose) *
T4a (Oral cavity): Tumor more than 4 cm in greatest dimension and more than 10 mm depth of invasion, or tumor invades through the cortical bone of the mandible or maxilla or involves the maxillary sinus, or invades the skin of the face*
T4b (Lip and oral cavity): Tumor invades masticator space, pterygoid plates or skull base, or encases internal carotid artery.

Note:

*Superficial erosion alone of bone/tooth socket by gingival primary is not sufficient to classify a tumor as T4a.

- Evaluation is based on the physical and imaging findings.
- For minor salivary gland tumors, this rule is applied, but the concept of DOI is excluded in T factor.

#### (2) Regional Lymph Node Metastasis

The classification and range of cervical lymph nodes are the same as described in the Rules Regarding Lymph Nodes by the Japan Society of Clinical Oncology (JSCO). Lymph node metastasis was evaluated according to the UICC classification (8th Edition). Internationally, the level classification system by ACHNSO based on the area of neck dissection is widely used, and the AAO-HNS classification, a fragmented version of the ACHNSO classification, has also been proposed.

1. Site
  Regional lymph node groups (JSCO), Level classification (AAO-HNS).
2. Number of metastasis
  Number of metastatic lymph nodes.
3. Size (< 3 < 6 <) cm
4. Clinical extranodal extension: cENE (−/ +)
  Adhesiveness (−/ +), Nerve involvement (−/ +).
5. N—Regional Lymph Nodes

Clinical N (cN).

NX: Regional lymph nodes cannot be assessed.
N0: No regional lymph node metastasis.
N1: Metastasis in a single ipsilateral lymph node, ≤ 3 cm in greatest dimension and cENE (−).
N2a: Metastasis in a single ipsilateral lymph node > 3 cm but ≤ 6 cm in greatest dimension and cENE (−).
N2b: Metastasis in multiple ipsilateral lymph nodes, none > 6 cm in greatest dimension and cENE (−).
N2c: Metastasis in bilateral or contralateral lymph nodes, none > 6 cm in greatest dimension and cENE (−).
N3a: Metastasis in a lymph node, > 6 cm in greatest dimension and cENE (−).
N3b: Metastasis in any node(s) and cENE ( +)*

Note:

*The presence of skin involvement or soft tissue invasion with deep fixation/tethering to underlying muscle or adjacent structures or clinical signs of nerve involvement is classified as clinical extranodal extension (cENE).

**Fig. 3:**
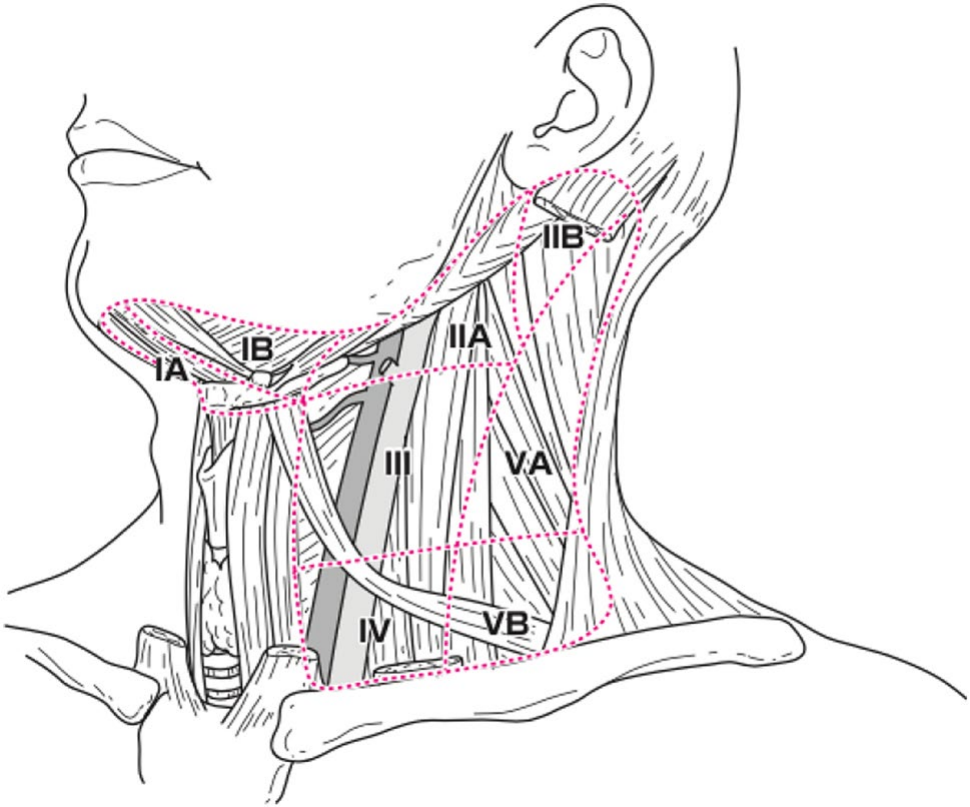
Classification of cervical lymph node levels

Level classification (Fig. 3).

Level IA: Submental lymph nodes.
Level IB: Submandibular lymph nodes.
Level IIA: Superior deep cervical lymph nodes (Jugulodigastric nodes) (anterior).
Level IIB: Superior deep cervical lymph nodes (Jugulodigastric nodes) (posterior).
Level III: Middle deep cervical lymph nodes (Jugulo-omohyoid nodes).
Level IV: Inferior deep cervical lymph nodes.
Level VA: Spinal accessory lymph nodes.
Level VB: Supraclavicular lymph nodes.

#### (3) M—distant metastasis

Distant metastases are evaluated according to the UICC classification (8th Edition).

MX: Presence of distant metastasis cannot be assessed.
M0: No distant metastasis.
M1: Distant metastasis.

- The category M1 may be further specified according to the following notation: Pulmonary (PUL), hepatic (HEP), osseous (OSS), lymph nodes (LYM), adrenal gland (ADR), brain (BRA), skin (SKI), and others (OTH).

#### (4) Stage (Table 1)

The clinical stage is determined according to the UICC classification (8th edition). The T, N, M factors and stages are recorded (Stage 0, I, II, III, IVA, IVB, and IVC).

- In the 1st edition of general rules for clinical and pathological studies on oral cancer, lower gingival cancer with invasion through the cortical bone is insufficient for T4a classification, but the tumor with invasion reaching the mandibular canal (MC) is classified as T4a [1, 2]. This classification has been supported by a study of 345 patients with lower gingival cancer [3]. The status of bone invasion in oral cancer should, therefore, be clarified. Bone invasion in oral cancer is categorized into three types: no bone invasion [BI (−)], bone invasion is absent or limited to cortical bone; medullary invasion [BI ( +)], invasion into cancellous bone is present, but does not extend into the MC; and MC invasion [MC ( +)], invasion extends into the MC.

**Table 1:** Staging

	N0	N1	N2	N3	M1
Tis	0				
T1	I	III	IVA	IVB	IVC
T2	II	III	IVA	IVB	IVC
T3	III	III	IVA	IVB	IVC
T4a	IVA	IVA	IVA	IVB	IVC
T4b	IVB	IVB	IVB	IVB	IVC

#### (5) Oral mucosal melanoma

Oral mucosal malignant melanoma is described separately because the classification is different. This classification applies only to oral malignant melanoma. Classification rules are based on the malignant melanoma of the head and neck and gastrointestinal tract.

There should be histological confirmation of the disease and division of cases by site. The evaluation methods in the TNM category are physical examination and diagnostic imaging. The description is based on the description of oral cancer.

pN0: Histological examination of regional lymphadenectomy specimens will ordinary include 6 or more lymph nodes. If the lymph nodes are negative, but the number ordinarily examined is not met, classify as pN0.

T—Primary Tumor.

TX: Primary tumor cannot be assessed.
T0: No evidence of primary tumor.
T3: Tumor limited to epithelium and/or submucosa (mucosal disease).
T4a: Tumor invades deep soft tissues, cartilage, bone, or overlying skin.
T4b: Tumor invades any of the following: brain, dura, skull base, lower cranial nerves (IX, X, XI, XII), masticator space, carotid artery, prevertebral space, mediastinal structures.

Note:

Mucosal melanoma is an aggressive tumor, therefore, T1 and T2 are omitted, as are stages I and II.

N—Regional Lymph Nodes.

NX: Regional lymph nodes cannot be assessed.
N0: No regional lymph node metastasis.
N1: Regional lymph node metastasis.

M—Distant Metastasis.

M0: No distant metastasis.
M1: Distant metastasis.

Stage.

Stages follow the UICC classification (8th edition).

#### (6) Multiple, Double, and Multiple Primary Cancers

1. Multiple oral cancers: The occurrence of two or more primary cancers fulfilling the following conditions:
  1. Cancer located at different sites according to the UICC classification
  2. Cancer located at the same but contralateral sites.
  3. Cancer located at ipsilateral sites, but not continuous, and clinically separated by 2.0 cm or more.
  4. Each lesion is histopathologically confirmed to be a carcinoma.
2. Double cancer: The concurrence of primary oral cancer with primary malignant tumors of other organs. If both multiple and double cancer are observed, they are expressed as multiple-double cancer.
3. Synchronous and metachronous cancer:
  1. Cancers diagnosed within 1 year of each other are defined as synchronous cancers.
  2. Cancers diagnosed at intervals of 1 year or longer are defined as metachronous cancers.
  3. If there are both synchronous and metachronous cancers, they are called synchronous/metachronous cancers.

Note:

(i) The organs affected by double cancers are indicated.
(ii) Whether the cancers are synchronous or metachronous is indicated.

e.g., Double cancers: Stomach (synchronous).

#### (7) Oral potentially malignant disorders (OPMDs)

Since oral cancers are often accompanied by oral potentially malignant disorders, such as leukoplakia, erythroplakia, and oral lichen planus, details including their site, size, surface properties, and number, as well as whether they are synchronous or metachronous are recorded.

#### (8) Lifestyle

Individual lifestyle is very significant as a risk factor for oral cancer. Smoking and drinking, in particular, are major risk factors for cancer. Therefore, the lifestyle needs to be recorded.

The presence or absence of preference history regarding smoking and drinking should be described. Furthermore, the amount of smoking is recorded according to the Brinkman index, and the amount of drinking is recorded according to the Sake index.

- Brinkman index = mean number of cigarettes smoked per day × number of smoking years
- Sake index = mean amount of drinking per day (in mL of sake) × number of drinking years

Scales for conversion into ml of Sake:

180 ml of sake = a large bottle of beer (633 mL) = 2 single glasses of whisky (70 mL) = 90 mL of shochu (distilled spirit) = 2 glasses of wine (220 mL).

### II. Recording of intraoperative findings and those on gross examination of the resected specimens

#### (1) Surgical procedure

The details entered into the operation record should be illustrated in detail. Whether or not reconstruction was performed, the construction procedure, intraoperative complications, radicality of the procedure, and the postoperative course should also be recorded.

##### Primary lesion

1. Tongue cancer:
  - partial tongue resection
  - unilateral resection of the movable part of the tongue
  - (semi)total resection of the movable part of the tongue
  - unilateral resection of the tongue
  - (sub)total tongue resection
2. Upper gingival and alveolus cancer/hard palate cancer:
  - local resection
  - partial maxillectomy
  - subtotal maxillectomy
  - total maxillectomy
  - extended total maxillectomy
  - skull base dissection
3. Lower gingival and alveolus cancer:
  - gingival resection
  - marginal mandibulectomy
  - segmental mandibulectomy
  - hemimandibulectomy
  - subtotal mandibulectomy
  - total mandibulectomy
4. Buccal mucosal cancer
  - partial (buccal mucosal) resection
  - combined resection
5. Floor of mouth cancer:
  - partial resection (of the floor of the mouth)
  - combined resection

Note:

If combined resection is performed, the resected parts, extent of resection, depth of resection, etc., should be recorded.

##### Cervical lymph nodes

1. Radical neck dissection (RND)
2. Modified radical neck dissection (MRND)
3. Selective neck dissection
  - Supraomohyoid neck dissection (SOHND)
  - Extended supraomohyoid neck dissection (ESOHND)
  - Suprahyoid neck dissection (SHND)

#### (2) Gross findings in resected specimens

##### Clinical information

1. Size
  Long diameter (mm) × Short diameter (mm) × Thickness (mm).
2. Lymph node metastasis
3. Preoperative therapy (−/ +).

##### Primary lesion

1. Location of the lesion.
2. Number and size of the lesions.
  Long diameter (mm) × Short diameter (mm) × Thickness (mm).
3. Depth of invasion (mm).
4. Macroscopic types
  Superficial type: Those primarily showing superficial growth.
  Exophytic type: Those primarily showing exophytic growth.
  Endophytic type: Those primarily showing endophytic growth.
5. Evaluation of margin status (−/ +)

##### Lymph node metastasis

1. Locations
  Lymph node groups (JSCO)/Level classification (AAO-HNS).
2. Number of metastatic lymph nodes
  Number of metastatic lymph nodes/number of resected lymph nodes.
3. Size (< 3 < 6 <) cm
4. Extranodal extension.
  cENE (−): no finding of clinical ENE.
  cENE ( +): finding of clinical ENE.
  Histological examination of a selective neck dissection specimen ordinarily includes 10 or more lymph nodes. Histological examination of a radical or modified radical neck dissection specimen ordinarily includes 15 or more lymph nodes. If the lymph nodes are negative, but the number ordinarily examined is not met, it is classified as pN0.

### III. Recording of pathological findings

#### (1) Clinical information

1. Surgical Procedure: (sub)total resection of the movable part of the tongue, marginal mandibulectomy, etc.
2. Size
  Long diameter × Short diameter × Thickness (mm).
3. Regional lymph node metastasis
  1. Size (< 3  < 6  <) cm
  2. Clinical extranodal extension: cENE.
    cENE (−): Clinically negative findings for extranodal extension.
    cENE (+): Clinically positive findings for extranodal extension.
4. Preoperative treatment (−/+)
  (−).
  (+) (contents of treatment).
  (Chemotherapy, Radiation therapy, etc.)

#### (2) Description of the primary tumor

1. Tumor location
2. Size and number of lesions
  Number of lesions: Single/Multiple.
  Long diameter × Short diameter × Thickness (mm).
3. Depth of invasion (DOI)
  DOI assesses the invasiveness of a carcinoma, regardless of any exophytic component. It measured by first finding the “horizon” of the basement membrane of the adjacent squamous mucosa.
4. Macroscopic type
  Superficial type.
  Exophytic type.
  Endophytic type.

#### (3) Pathological findings

##### Histological classification and grading

1. Histological classification
  -    Epithelial tumors
      Carcinoma in situ 8070/2.
      Carcinoma 8010/3.
    Squamous cell carcinoma 8070/3.
      Basaloid squamous cell carcinoma 8083/3.
      Spindle cell squamous cell carcinoma 8074/3.
      Adenosquamous carcinoma 8560/3.
      Carcinoma cuniculatum 8051/3.
      Verrucous squamous cell carcinoma 8051/3.
      Lymphoepithelial carcinoma 8082/3.
      Papillary squamous cell carcinoma 8052/3.
      Acantholytic squamous cell carcinoma 8075/3.
    Adenocarcinoma 8140/3.
      Mucoepidermoid carcinoma 8430/3.
      Adenoid cystic carcinoma 8200/3.
      Polymorphous adenocarcinoma 8525/3.
      Clear cell carcinoma 8310/3.
      Basal cell adenocarcinoma 8147/3.
      Carcinoma ex pleomorphic adenoma 8941/3.
    (b)    Malignant odontogenic tumors
      Ameloblastic carcinoma 9270/3.
      NOS Primary intraosseous carcinoma, NOS 9270/3.
      Sclerosing odontogenic carcinoma 9270/3.
      Clear cell odontogenic carcinoma 9341/3.
      Ghost cell odontogenic carcinoma 9302/3.
      Odontogenic carcinosarcoma 9342/3.
      Odontogenic sarcoma 9330/3.
  2. Histopathological grading
  GX: Grade of differentiation cannot be assessed.
  G1: Well differentiated.
  G2: Moderately differentiated.
  G3: Poorly differentiated.
  G4: Undifferentiated.

Note:

Grades 3 and 4 can be combined in some circumstances as ‘G3–4, poorly differentiated or undifferentiated’. The main primary tumor may be recorded and may describe mixed lesions as’G1 > G3, G1 + G3’.

##### pTNM pathological classification

1. pT—Primary tumor
  The pT categories correspond to the clinical T categories.
2. pN—Regional lymph node

pNX: Regional lymph node cannot be assessed.
pN0: No regional lymph node metastasis.
pN1: Metastasis in a single ipsilateral lymph node, 3 cm or less in greatest dimension without extranodal extension.
pN2: Metastasis described as:
pN2a: Metastasis in a single ipsilateral lymph node, 3 cm or less in greatest dimension with extranodal extension or, more than 3 cm but not more than 6 cm in greatest dimension without extranodal extension.
pN2b: Metastasis in multiple ipsilateral lymph nodes, none more than 6 cm in greatest dimension, without extranodal extension.
pN2c: Metastasis in bilateral or contralateral lymph nodes, none more than 6 cm in greatest dimension, without extranodal extension.
pN3a: Metastasis in a lymph node more than 6 cm in greatest dimension without extranodal extension.
pN3b: Metastasis in a lymph node more than 3 cm in greatest dimension with extranodal extension or, multiple ipsilateral, or any contralateral or bilateral node(s) with extranodal extension.

Note:

(i) Histological examination of a selective neck dissection specimen ordinarily includes 10 or more lymph nodes. Histological examination of a radical or modified radical neck dissection specimen ordinarily includes 15 or more lymph nodes. If the lymph nodes are negative, but the number ordinarily examined is not met, it is classified as pN0.
(ii) Midline nodes are considered ipsilateral nodes.
(iii) Direct invasion of the primary tumor to lymph nodes is classified as lymph node metastasis.
(iv) When size is a criterion for pN classification, measurement is made of the metastasis, not of the entire lymph node. The measurement should be that of the largest dimension of the tumor. The conglomerate of lymph nodes should be considered as the individual lymph node.
3. pM—Distant metastasis
  pM1: Distant metastasis microscopically confirmed.
  Note (1) pM0 and pMX are not valid categories.
  Note (2) Metastasis in any lymph node other than regional is classified as M1 and metastatic organs are described with the following notation: Lymph node (LYM), Skin (SKI), Pulmonary (PUL), Bone marrow (BAR), Osseous (OSS), Brain (BRA), etc.
4. Stage

##### Margin status

1. Horizontal margin: HM
  HMX: Involvement of the horizontal margin cannot be assessed.
  HM0: No involvement of the horizontal margin.
  HM1: Involvement of the horizontal margin.
2. Vertical margin: VM
  VMX: Involvement of the vertical margin cannot be assessed.
  VM0: No involvement of the vertical margin.
  VM1: Involvement of the vertical margin.
    Note:
  (i): The prefix p is used when margin status is assessed histopathologically.
  (ii): For horizontal (surface mucosa) margin status, a description of the dysplasia grade or Tis component is added along with the site (desirable to describe the specimen number). For those that cannot be confirmed, record the distance to the tumor.
3. Residual tumor
  RX: Presence of residual tumor cannot be assessed.
  R0: No residual tumor.
  R1: Microscopic residual tumor.
  R2: Macroscopic residual tumor.

##### Histological descriptors

The histological descriptors are graded according to the need for description.

Grade A is good evidence to recommend to describe.

Grade B is fair supporting evidence to describe.

1. Vascular invasion (Ly, V)

- Lymphatic invasion (Ly) (Grade A)
  LyX: Lymphatic invasion cannot be assessed.
  Ly0: No lymphatic invasion.
  Ly1: Lymphatic invasion.
  Ly1a: Minimal lymphatic invasion.
  Ly1b: Moderate lymphatic invasion.
  Ly1c: Marked lymphatic invasion.

Note:

If immunostaining has been used for the evaluation of lymphatic vessel invasion, it must be recorded, e.g., Ly1a (D2-40).

(b) Venous invasion (V) (Grade A)
  VX: Venous invasion cannot be assessed.
  V0: No venous invasion.
  V1: Venous invasion.
  V1a: Minimal venous invasion.
  V1b: Moderate venous invasion.
  V1c: Marked venous invasion.
  V2: Macroscopic venous invasion.

Note:

If immunostaining or elastic fiber staining has been used for the evaluation of venous invasion, it must be recorded, e.g., V1b (EVG).

2. Perineural invasion (Pn) (Grade A)
  PnX: Perineural invasion cannot be assessed.
  Pn0: No perineural invasion.
  Pn1: Perineural invasion.
  Pn1a: Minimal perineural invasion.
  Pn1b: Moderate perineural invasion.
  Pn1c: Marked perineural invasion.

Note:

If immunostaining has been used for the evaluation of perineural invasion, it must be recorded, e.g., Pn1a (S-100).

3. Mode of invasion (YK classification) (Grade B)
  YK-1: Well defined borderline.
  YK-2: Cords, less marked borderline.
  YK-3: Groups of cells, no distinct borderline.
  YK-4C: Diffuse invasion, cord-like invasion.
  YK-4D: Diffuse invasion, diffuse type invasion.

4. Regional lymph node metastasis (Grade A)
  Location (Lymph node groups).
  Number (Number of metastatic lymph nodes/number of resected lymph nodes).
  Size (< 3 < 6 <) cm.
  Extranodal extension (ENE).
  ENE (−): Negative findings for extranodal extension.
  ENE ( +): Positive findings for extranodal extension.
5. Histological evaluation of therapeutic effect

In examining patients after preoperative treatment, specimens of the grossly estimated lesion must be prepared as much as possible, and the state of the remaining tumor must be evaluated histologically.

Grade 0: Ineffective.
  No therapeutic effect is noted in cancer tissue or cancer cells.
Grade 1: Slightly effective.
  Some degenerative change is noted in cancer tissue/cells, but cancer cells considered to be capable of proliferation (those showing eosinophilic cytoplasm with vacuolation and enlargement of the nucleus are also included) occupy 1/3 or more of the cancer in a tissue section.
Grade 1a: Very slightly effective.
  Cancer cells considered to be “capable of proliferation” occupy 2/3rd or more of the cancer.
Grade 1b: Mildly effective.
  Cancer cells considered to be “capable of proliferation” occupy 1/3rd or more but less than 2/3rd of the cancer.
Grade 2: Moderately effective.
  Cancer cells considered to be “capable of proliferation” occupy less than 1/3rd of the cancer, and those showing a tendency toward nuclear disintegration are dominant.
Grade 3: Markedly effective.
  No cancer cell considered to be “capable of proliferation” is observed, and all cancer cells show a tendency toward nuclear disintegration, or only a trace of cancer is noted.
  If a part clearly judged to be a focus of reproliferation is noted in a treated cancer focus, the entry “evidence of reproliferation ( +)” should be made after the judgment.

Note:

(i) If radiation therapy or chemotherapy has been conducted for oral cancer, conditions of treatment including the dose of radiation, irradiation method, kinds, doses, and administration methods of the drugs used, and time from the last treatment to resection of the lesion should be recorded.
(ii) This criterion is used for the primary lesion of the surgical material. For lymph node dissection specimens, describe any evidence of cancer cell disappearance, necrosis, or degeneration. For biopsy material, only histological findings should be included.

### IV. Other Treatments and Clinical Evaluation of the Therapeutic Effect

#### (1) Radiation Therapy

##### Basic entry items

1. Objective of irradiation
  definitive, palliative, preoperative, postoperative, preventive, etc.
2. Presence or absence of concomitant treatments
  irradiation alone, concomitant treatment performed (surgery, chemotherapy, etc.)
3. Degree of completion of radiation therapy
  completed (no interruption), completed (with interruption), not completed as planned.
4. Irradiation methods and treatment assessment (detailed entry items are described below).

##### External irradiation

1. Radiation type, instrument, and energy
  X-ray (Linac, CyberKnife, MV), electron beam (MeV), proton beam, heavy particle beam, etc.
2. Clinical target volume (CTV)
  primary lesion, cervical lymph nodes (left or right, levels), distant metastases (sites).
3. Irradiation methods
  one field irradiation (anterior, posterior, others), two-field irradiation (left-right, anterior-posterior, diagonal, others), three-dimensional conformal radiotherapy (3D-CRT), intensity-modulated radiotherapy (IMRT), stereotactic body radiation therapy (SDRT), others. If 2 or 3 sites are treated, treatment for each site is recorded separately. If the irradiation method has been changed, the objective of the change (reduction in the irradiation field, change in the junction line, protection of the spinal cord, others) and the dose at the change are recorded.
4. Planning of radiation therapy
  radiation therapy planning device, dose-volume histogram (DVH).
5. Use of compensatory instruments, bolus or fixation instruments
  physical or dynamic wedge, shell, bite block, others.
6. Radiation dose
  Dose per fraction (Gy), total dose (Gy), number of fractions, number of irradiations per day, irradiation intervals, irradiation period, completed or not completed. If 2 or 3 sites are treated, treatment for each site is recorded separately.
  E.g., 60 Gy/30 fr/43 days (2009.7.6—8.17).
7. Dose for organs at risk
  brain, eye (lens), spinal cord, salivary gland, others.

##### Brachytherapy

1. Types, high or low dose rate, and shape of radiation source
2. Irradiation dose

Target dose, number of irradiations, time of irradiation (time at the beginning, time at the end), dose rate (dose/hour), radioactivity of the source during use (MBq).

3. Relationships of reference points or planes for dose evaluation with tumor and radiation source.
4. Dose calculation method
  radiation therapy planning device.
5. Doses for organs at risk.

##### Treatment assessment

1. Effects of irradiation
  The therapeutic effects shortly after, 1 month after, and 3 months after irradiation are recorded.
2. Acute adverse events
  The severest organ or tissue damage, signs and symptoms, and the period of their observation are recorded, according to the latest version of CTCAE.
3. Late adverse events
  Adverse events that occur more than 91 days after the beginning of irradiation and are considered to be related to irradiation are recorded.

#### (2) Chemotherapy

With regard to the administration of chemotherapy using cytotoxic agents, molecular targeting drug, or immune checkpoint inhibitor, the following items should be included in the medical record:

##### Before treatment: description of the treatment plan

1. Patient’s data for determination of the treatment dose
  Performance status.
  Body surface area.
  Renal function and liver function.
2. Information for planned chemotherapy
  Treatment regimen.
  Purpose of treatment.
  Administration dose, route, and schedule for each drug.
3. Information for concomitant treatment
  If chemotherapy will be administered combined with local treatment, its details of (dose, timing, and purpose) should also be recorded.
4. Identification of lesions to be evaluated

Assessment of the change in tumor burden is an important feature of the clinical evaluation of cancer therapeutics. As baseline data, the target and non-target lesions should be identified before starting treatment, according to the latest Response Evaluation Criteria in Solid Tumors (RECIST) [4] guidelines at that time.

##### During and after treatment: description of the clinical course

1. Evaluation of adverse events caused by chemotherapy
  Assessment of the toxicity caused by chemotherapy is important for the adjustment of treatment plan and patient safety. Adverse events after the administration of chemotherapy should be evaluated according to the latest Common Terminology Criteria for Adverse Events (CTCAE) [5] at that time, including the causal relationship with chemotherapy.
2. Evaluation of therapeutic effect and decision of treatment continuation
  The therapeutic effect is evaluated by changes in the lesions identified at the baseline.
  As a note, RECIST [4] is just a criterion for therapeutic effect, not an absolute indicator in clinical practice. The decision for treatment continuation should be made with comprehensive consideration of the clinical course or patient condition.

### V. Therapeutic Results

1. Number of patients
  1. Total number of outpatients
  2. Total number of hospitalized patients
2. Multiple oral cancer, double cancer
3. Main treatment and adjuvant therapy
  1. Surgical treatment
  2. Radiotherapy
  3. Chemotherapy
  4. Other treatments
4. Objective of Treatment
  definitive, palliative, preoperative, neoadjuvant, adjuvant, etc.
5. Evaluation of postoperative mastication (swallowing, vocalization) function
  1. Evaluation of speech function: clarity of single syllable pronunciation, clarity of speech
  2. Evaluation of eating function:
    1. Occlusion/mastication: questionnaire survey using Yamamoto’s bite scale, color gum test, dental prescale test
    2. Swallowing: Water swallowing test, videofluorography (VF), videoendoscopy (VE), cervical auscultation
6. Analysis of treatment results
  Results of treatment are based on the Japanese Cancer Therapy Society, Joint Committee on Cancer Therapy Edited by the Japan Cancer Therapy Society/Cancer Regulations, and UICC general rules. When displaying the results, it is necessary to clearly specify the definition of the target case and the treatment of deaths from other diseases, untraceable cases, and cases with unknown causes.
7. Object case
  All cases should be histologically confirmed. Cases for which a histological definitive diagnosis cannot be obtained due to various circumstances and clinically apparent cancer are necessary. The setting of the object case is most important for calculating the survival rate.
  1. Primary or untreated cases:
    Patients who have not been treated for the cancer at another facility until the first consultation. Test resection for diagnosis is not treated.
  2. Secondary or treated cases
    Cases for which the treatment has already been started or completed at the first consultation for the cancer.
  3. Confirmed cases
    The definition can be freely determined by the researcher depending on the purpose of the research. However, the definition must be clearly stated. In general, it refers to cases in which all other disease cases have been excluded from cases with obvious death from other diseases and discontinued treatment. Untraceable cases are not excluded.
8. Outcome
  1. Tracing origin
    Basically, the date of treatment is determined, but the patient who has been treated may be the treatment start date. Untreated patients are the dates when they decide not to receive treatment. For oral cancer, the first consultation day is often the same as the treatment policy decision date.
  2. Tracking date
    Specific date law (usually December 31) or anniversary law (usually birthday). Untracked patients must be minimized.
  3. Survival state
    1. Survivors: date of confirmation of being alive
    2. Those that have died: date of death
    3. Those lost to follow-up: last date of confirmation of being alive
  4. Causes of death
    1. Treatment-related death (death due to surgical treatment, chemotherapy, radiation therapy, etc.)
    2. Death due to oral cancer (death due to primary disease)
    3. Death due to another malignant disease: record the tumor name
    4. Death due to another disease: record the disease name
    5. Death due to an accident: including suicide
    6. Cause of death unclear
    7. Autopsy performed or not performed
9. Method for the Calculation of Long-Term Follow-up Results

It is desirable to show various survival rates as cumulative survival rates from the day of the beginning the treatment, and this is usually done using the Kaplan–Meier method. Tests to examine the significance of differences in the survival rate include the generalized Wilcoxon test, Mantel–Haenszel test, log-rank test, and Cox–Mantel test.
